# SIDS–CDF Hypothesis Revisited: Cause vs. Contributing Factors

**DOI:** 10.3389/fneur.2016.00244

**Published:** 2017-01-16

**Authors:** Pontus M. A. Siren

**Affiliations:** ^1^Independent Researcher, Ridley Park, Singapore

**Keywords:** SIDS, diaphragm, respiratory failure, hyperthermia, magnesium deficiency

## Abstract

The sudden infant death syndrome (SIDS)–critical diaphragm failure (CDF) hypothesis was first published by Siren and Siren in 2011 (1). Since its publication, the hypothesis has continued to generate interest and several colleagues have contributed perspectives and insights to it (2–5). The basic premise of the hypothesis is that the diaphragm is a vital organ that must continuously generate adequate force to maintain ventilation, and that CDF is a terminal event and the cause of death in SIDS. I have argued in two follow-up articles that all SIDS factors either increase the workload of the respiratory muscles, the diaphragm being the primary muscle affected, or reduce its force generating capacity (6, 7). The SIDS–CDF hypothesis posits that SIDS has many contributing factors but only one cause, namely, the failure of the vital respiratory pump. There are several known SIDS factors, such as the prone sleeping position, non-lethal infections, deep sleep, gestational prematurity, low birth weight, cigarette smoke, male gender, and altitude, but of these, some such as the prone sleeping position more significantly both impact diaphragm function and correlate with SIDS. However, SIDS cases are multifactorial and as such can be caused by different combinations of factors. An infection combined with a prone sleeping position and elevated room temperature could lead to SIDS, whereas in other circumstances, low birth weight, cigarette smoke, prone sleeping position, and altitude could result in CDF and SIDS. The SIDS–CDF hypothesis also posits that SIDS does not have a congenital or genetic origin, and that efforts to identify significant genetic anomalies in SIDS victims are unlikely to be successful (8–11).

This short review has two purposes. The first is to examine two SIDS risk factors in the context of the SIDS–CDF hypothesis that have not been addressed in previous articles, namely, magnesium deficiency and hyperthermia. Second, this review is an attempt to raise awareness of the research community to the possible role of the vital respiratory muscles in etiology of the syndrome. As I reiterated in a recent article, the possibility that SIDS is caused by critical weakness of the diaphragm has been largely ignored. It is telling that as of November 2016, out of approximately 11,000 SIDS articles in PubMed, only 50 articles contained the search words “SIDS and diaphragm,” and only a few of those actually address the role of the vital respiratory muscle in SIDS. At the end of the commentary, I will suggest avenues for future research.

In 1972, Joan Caddell advanced a hypothesis in *The Lancet* arguing that magnesium deficiency is the cause of death in SIDS (12). Between 1972 and 2001, Caddell and others attempted to provide experimental evidence between magnesium deficiency and SIDS, but the hypothesis remains neither proven nor disproven (13). Systemic magnesium levels are notoriously difficult to measure accurately, and studies on magnesium deficiency in SIDS victims are inconclusive (14, 15). A causal mechanism was never established, although magnesium deficiency shock and compromised thermoregulation were proposed as possible culprits (16, 17). However, Caddell’s hypothesis prompted several interesting studies. She asserted that ethnic groups with low SIDS rates (at or below 1.2 per 1,000 live births) have rich dietary sources of magnesium, while those with SIDS rates exceeding 5.0 typically have magnesium poor diets (17), and while the evidence for this is circumstantial, there are two other population level studies that warrant our closer attention. Following the publication of Caddell’s hypothesis, Swift and Emery suggested that “the best way to test Caddell’s hypothesis would be to attempt a correlation of the incidence of unexpected death to areas where there is a deprivation of magnesium in the water-supply” (18). Two studies, conducted some 30 years apart in USA and Taiwan, do exactly that. Despite the different population base and geography, the studies reach strikingly similar conclusions about the relationship between magnesium in municipal drinking water and the incidence of SIDS.

The first study was published in *The Lancet* in 1973 and was based on data from the California State Department of Public Health that provided ranges of magnesium and calcium concentrations in county water supplies. The authors concluded that “the median maximum magnesium concentration is lower in counties with higher rates of S.U.D. [sudden unexpected infant death].” The authors note that the study has several limitations such as the counties having large ranges for magnesium and the strong negative correlation of magnesium and calcium concentrations to overall infant mortality (19). By itself, the study provides interesting but insufficient data to suggest that magnesium levels in municipal water affect SIDS rates.

However, a similar, but far more robust, study was conducted in Taiwan in 2005, which reached similar conclusions. The study by Chiu and colleagues used data from the Taiwan Water Supply Corporation and mapped all SIDS death (501 cases) from 1988 to 1997 to controls who died from other causes (20). The mean magnesium concentration in municipal water was 9.69 mg/l for SIDS cases and 11.46 for controls. The authors note: “the group with the highest magnesium levels (>14.1 mg/l) had an OR [odds ratio] which remained significantly less than 1.0 (0.70, 95% CI = 0.51–0.97). In addition, there was a significant trend toward a decreased SIDS risk with increasing magnesium levels in drinking water (*X*^2^ for linear trend = 12.83, *p* < 0.05).” The authors conclude that there seems to be a significant protective effect of magnesium intake from drinking water against SIDS. They further observe that: “the fact that a significantly protective effect of magnesium intake *via* drinking water was found in the group with the highest levels of intake suggested that only subjects with magnesium intake *via* drinking water above a certain level receive a beneficial effect on their risk of SIDS.” The authors also address the question of how the relatively small intake on magnesium from drinking water can significantly affect the amount of magnesium in the body and point to research on magnesium absorption from drinking water that support this hypothesis (21). Any study of this nature has limitations, but due to the sophisticated health care and administrative system in Taiwan and the rigorous categorization of causes of death, the authors argue that these have been appropriately mitigated. The same research group has established correlations between magnesium levels in municipal water and the occurrence of various types of cancers, hypertension, and diabetes. No causal mechanism for the role of magnesium in SIDS is offered.

The studies by Chiu and his team and the earlier work by Godwin and Brown provide enough robust data to warrant an explanation, and I will address how the SIDS–CDF hypothesis would explain the data. The hypothesis posits that SIDS is caused by factors that either increase the respiratory workload of the diaphragm or decrease its force generating capacity. It is well known that magnesium deficiency can cause significant muscle weakness in humans (22, 23), and as Caddell points out, “reduced muscle power is a major clinical finding in magnesium deficient children” (24). Dhingra and colleagues showed a direct correlation between hypomagnesemia and respiratory muscle function in humans (25), and Stendig-Lindberg and colleagues demonstrated that maximum isometric voluntary contraction force was significantly weaker in hypomagnesaemic subjects (26, 27). These and other recent studies (28) show that magnesium deficiency directly and significantly impacts muscle strength in general and respiratory muscle strength specifically. As Matias and colleagues explain, magnesium deficiency has a significant effect on muscle performance, probably due to the key role of magnesium in energetic metabolism, transmembrane transport, muscle contraction, and on the cellular level on Na-K ATPase, Na–K–Cl co-transport, K channels, charge screening, and permeability effects on membranes (29). In this context, it is important to note that in terms of contractile properties and fatigue, the diaphragm behaves like other skeletal muscles (30).

At least two studies show that SIDS victims and near-miss infants have abnormalities in muscle tone. In 1976, the results from a large study containing 1,553 infants and 12 unexpected deaths were published. The study used the Graham–Rosenblith Scales behavioral examination to measure muscle strength and co-ordination. Of the 12 likely SIDS cases, all but 1 had no unusual findings on muscle tonicity. The infants displayed marked head lag, poor tonicity, moderate trembling or shaking, arching, and hyperactivity (31). The authors associated this with central nervous system involvement, but it is useful to consider other possible explanations. As Flink notes, important symptoms of magnesium deficiency are “neuromuscular hyperactivity including tremor, myoclonic jerks [and] convulsions” (32). Similarly, Wong and Teh reported on 13 cases of convulsions or tremors in children (aged 1 day to 14 months) with hypomagnesemia. The authors note that on recovery, either spontaneously or after administration of magnesium, serum magnesium returned to normal levels, and that it seems likely that hypomagnesemia caused the tremors or convulsions (33). Another SIDS study to consider in this context is the systematic neurologic examination of 41 near-miss infants, 7 normal siblings of SIDS victims, and 21 normal control infants conducted by the Sudden Death Research Project at Stanford University School of Medicine. The study found consistent abnormalities of muscle tone in the near-miss infants who were under 3 months of age (34). The evidence discussed above helps put the municipal water/magnesium studies in context and suggests that SIDS victims and near-miss infants have compromised muscle function (whether or not associated with hypomagnesemia). This fact by itself is significant in the context of the SIDS–CDF hypothesis.

Magnesium is a central element with multiple roles in human biology, and as Baaij and colleagues point out, “magnesium is an essential ion for health and it plays an important role in the physiological function of the brain, heart, and skeletal muscles” (35). In light of the existing evidence, it would be presumptuous to discuss its potential role in SIDS but tentatively. Still, the two population-level studies and the clinical results from SIDS and near-miss infants provide intriguing data on the potential role of magnesium and muscle weakness in the etiology of SIDS. The critical role of magnesium in muscle function is well established, and compelling data suggest that SIDS victims have abnormal muscle tonicity. Combined with the existing evidence regarding the possible role of respiratory muscles in SIDS, it seems obvious that the role of the diaphragm in SIDS should be rigorously investigated.

Hyperthermia is the other factor that I would like to discuss in the context of the SIDS–CDF hypothesis. Dallas first suggested that overheating might contribute to SIDS in 1974 but presented no evidence (36). Several authors have since argued that hyperthermia is significantly associated with SIDS (37–39). In 1984, Stanton reported that of the 34 SIDS victims: “19 babies were unusually hot or sweating when found dead; 14 died in an unusually warm environment; 17 had evidence of a terminal infective illness; and 24 were excessively clothed or overwrapped … In 6 of 15 babies (40%) whose rectal temperature was recorded after death, the temperature was above 37°C, the highest being 42°C” (40). In 1990, Fleming and colleagues reported that, “overheating and the prone position are *independently* associated with an increased risk of sudden unexpected infant death, particularly in infants aged more than 70 days” (41), and *The Lancet* published an editorial titled *Prone, Hot and Dead* that discussed link between hyperthermia and SIDS the same year. The editors note the pathological findings are non-specific and that no mode of action has been established. Interestingly, they also highlight that researchers have observed that: “environmental temperature of healthy infants increased respiratory movement, a finding that suggests an effect on the respiratory control system.” The editorial suggests that a possible mechanism is: “[hyperthermia] together with an additional factor—for example, a viral infection—is the stimulus for a further increase in metabolic rate, with subsequent loss of respiratory control. This view accords with several other observations that symptoms of illness, especially of respiratory infection, are present in many infants who die from SIDS.” (42).

However, while there is robust evidence linking hyperthermia to SIDS, no causal mechanism has been established. The elevated temperatures observed in SIDS victims are regularly observed in infants who do not succumb to the syndrome, and importantly, many SIDS victims show no evidence of hyperthermia. Recent research found no significant expression of heat-shock proteins (HSP27 and HSP70), thereby suggesting that hyperthermia is not a primary causal factor in SIDS (43). As with many SIDS risk factors, hyperthermia is a paradox. There is compelling epidemiological and population data to suggest that at least in some cases, hyperthermia is a significant risk factor for SIDS, but at the same time, it is highly unlikely that hyperthermia is the cause of SIDS.

To explain this paradox, it is useful to recall the observation by *The Lancet* editors regarding how higher environmental temperature increases the respiratory movement in healthy infants. Indeed, it has been known since 1905 that hyperthermia increases the ventilatory workload (44). More recently, Maskrey observed that: “high body temperature causes an increased ventilation (chiefly through increased frequency of breathing) *via* a descending drive from thermoreceptors and thermoregulatory integrating centers in the hypothalamus” (45). In a comprehensive recent review, Tsuji and colleagues again show that hyperthermia can induce hyperventilation in humans (46). The authors point out that: “during passive heating at rest, elevation of body core temperature leads to increased ventilation independently of metabolic factors, resulting in a reduction of arterial CO_2_ pressure (hypercapnia).” The critical threshold for the initiation of hyperventilation for passive heating at rest is when body core temperature reaches a critical threshold of ~38.5°C. As discussed in the beginning of this review, the SIDS–CDF hypothesis argues that any factor that either increases the respiratory workload or decreases the force generating capacity of the respiratory muscles can contribute to SIDS. Hyperthermia is known to increase the respiratory workload, and combined with other factors such as non-lethal infections and the prone sleeping position, it could, in some circumstances, push the diaphragm over its endurance threshold.

I have reviewed evidence suggesting that magnesium deficiency and hyperthermia are contributing, but not causal, factors in SIDS, and that by themselves, neither magnesium deficiency nor hyperthermia lead to CDF. As noted earlier, both magnesium deficiency and hyperthermia affect multiple physiological functions, and we must be cautious in drawing conclusions about their role in SIDS. Suffice it to say that the SIDS–CDF hypothesis offers a parsimonious causal mechanism for the large body of data on magnesium deficiency, hyperthermia, and SIDS.

A central challenge of SIDS research is that is it very difficult to design experiments that mimic the syndrome. The underlying reason is that experiments by their very nature seek to isolate variables and SIDS is multifactorial. No single factor is “the cause” of SIDS, but many factors can contribute to respiratory muscle failure, often in different configurations. We know that severe respiratory muscle weakness can lead to death in adults; it remains to be determined if SIDS victims succumb to respiratory muscle weakness. To test the SIDS–CDF hypothesis, experiments should focus on the diaphragm and its vulnerabilities, and we should seek to develop experimental protocols to determine if induced and progressive respiratory muscle weakness in experimental animals replicates the pathophysical outcomes of SIDS. We should also seek identify possible molecular markers from the diaphragms of adults who have died of respiratory muscle failure and should actively leverage the extensive research by Supinski and Callahan into the molecular pathways of infection induced respiratory muscle weakness (47, 48). The respiratory pump is as vital as the heart, but very little experimental research has been conducted on the possible role of the diaphragm in SIDS. The purpose of this article has been, in part, to inspire that research.

## Author Contributions

The author confirms being the sole contributor of this work and approved it for publication.

## Conflict of Interest Statement

The author declares that the research was conducted in the absence of any commercial or financial relationships that could be construed as a potential conflict of interest.

## References

[1] Siren PMA Siren MJ. Critical diaphragm failure in sudden infant death syndrome. Ups J Med Sci (2011) 116:115–23. 10.3109/03009734.2010.548011 21222555 PMC3078540

[2] Eisenhut M Features of diaphragmatic myositis in a case of sudden infant death. Ups J Med Sci (2011) 116:220 10.3109/03009734.2011.588347 21671726 PMC3128728

[3] Szczesny P Poznanski J Paczek L Zielenkiewicz P Hypophosphatemia and sudden infant death syndrome (SIDS) – is ATP the link? Ups J Med Sci (2014) 119:55–6. 10.3109/03009734.2013.849317 24151935 PMC3916719

[4] Goldwater PN. A perspective on SIDS pathogenesis. The hypotheses: plausibility and evidence. BMC Med (2011) 9:64. 10.1186/1741-7015-9-64 21619576 PMC3127778

[5] Van Kempen TA Deixler E Crook MA Hypophosphatemia as a key factor in sudden infant death syndrome (SIDS)? Ups J Med Sci (2013) 118:143–4. 10.3109/03009734.2013.781252 23570456 PMC3633331

[6] Siren PMA The SIDS – critical diaphragm failure hypothesis revisited. Ups J Med Sci (2013) 118:62–4. 10.3109/03009734.2012.744373 23153369 PMC3572675

[7] Siren PMA The SIDS – critical diaphragm failure hypothesis revisited: explaining hypoxia in SIDS. Ups J Med Sci (2016) 121:199–201. 10.1080/03009734.2016.1176972 27460606 PMC4967269

[8] Paterson DS. Serotonin gene variants are unlikely to play a significant role in the pathogenesis of the sudden infant death syndrome. Respir Physiol Neurobiol (2013) 189:301–14. 10.1016/j.resp.2013.07.001 23851109 PMC3812255

[9] Rognum IJ Tran H Haas EA Hyland K Paterson DS Haynes RL Serotonin metabolites in the cerebrospinal fluid in sudden infant death syndrome. J Neuropathol Exp Neurol (2014) 73:115–22. 10.1097/NEN.0000000000000034 24423636 PMC4287961

[10] Highet AR Gibson CS Goldwater PN. CD14 (C-260T) polymorphism is not associated with sudden infant death syndrome (SIDS) in a large South Australian cohort. Innate Immun (2011) 17:321–6. 10.1177/1753425910369272 20472613

[11] Studer J Bartsch C Haas C. Tyrosine hydroxylase TH01 9.3 allele in the occurrence of sudden infant death syndrome in Swiss Caucasians. J Forensic Sci (2014) 59:1650–3. 10.1111/1556-4029.12526 24975687

[12] Caddell JL Magnesium deprivation in sudden unexpected infant death. Lancet (1972) 2(7771):258–62. 10.1016/S0140-6736(72)91690-X 4114510

[13] Caddell JL The apparent impact of gestational magnesium (Mg) deficiency on the sudden infant death syndrome (SIDS). Magnes Res (2001) 14(4):291–303. 11794637

[14] Chipperfield B Chipperfield JR Cot deaths and mineral salts. Lancet (1979) 1(8109):220 10.1016/S0140-6736(79)90627-5 84247

[15] Erickson MM Poklis A Gantner GE Dickinson AW Hillman LS Tissue mineral levels in victims of sudden infant death syndrome II. Essential minerals: copper, zinc, calcium, and magnesium. Pediatr Res (1983) 17(10):784–7. 10.1203/00006450-198310000-00003 6634242

[16] Durlach J Durlach V Rayssiguier Y Ricquier D Goubern M Bertin R Magnesium and thermoregulation. I. Newborn and infant. Is sudden infant death syndrome a magnesium-dependent disease of the transition from chemical to physical thermoregulation? Magnes Res (1991) 4(3–4):137–52. 1799550

[17] Caddell JL. A triple-risk model for the sudden infant death syndrome (SIDS) and the apparent life-threatening episode (ALTE): the stressed magnesium deficient weanling rat. Magnes Res (2001) 14(3):227–38. 11599557

[18] Swift PG Emery JL Magnesium and sudden unexpected infant death. Lancet (1972) 2(7782):871 10.1016/S0140-6736(72)92227-1 4116568

[19] Godwin JD Brown C Magnesium and sudden unexpected infant death. Lancet (1973) 1(7813):1176 10.1016/S0140-6736(73)91166-5 4123554

[20] Chiu HF Chen CC Tsai SS Wu TN Yang CY. Relationship between magnesium levels in drinking water and sudden infant death syndrome. Magnes Res (2005) 18(1):12–8. 15945612

[21] Marx A Neutra RR. Magnesium in drinking water and ischemic heart disease. Epidemiol Rev (1997) 19(2):258–72. 10.1093/oxfordjournals.epirev.a017957 9494787

[22] Brautbar N Carpenter C. Skeletal myopathy and magnesium depletion: cellular mechanisms. Magnesium (1984) 3(2):57–62. 6503356

[23] Dralle D Bödeker RH. Serum magnesium level and sleep behavior of newborn infants. Eur J Pediatr (1980) 134(3):239–43. 10.1007/BF00441479 7428773

[24] Caddell JL. Magnesium deficiency promotes muscle weakness, contributing to the risk of sudden infant death (SIDS) in infants sleeping prone. Magnes Res (2001) 14(1–2):39–50. 11300621

[25] Dhingra S Solven F Wilson A McCarthy DS Hypomagnesemia and respiratory muscle power. Am Rev Respir Dis (1984) 129(3):497–8. 6703506 10.1164/arrd.1984.129.3.497

[26] Stendig-Lindberg G Is physical working capacity determined by optimal magnesium concentration? J Basic Clin Physiol Pharmacol (1992) 3(2):139–51. 1295571 10.1515/jbcpp.1992.3.2.139

[27] Stendig-Lindberg G Bergström J Hultman E. Hypomagnesaemia and muscle electrolytes and metabolites. Acta Med Scand (1977) 201(4):273–80. 851037 10.1111/j.0954-6820.1977.tb15699.x

[28] Veronese N Berton L Carraro S Bolzetta F De Rui M Perissinotto E Effect of oral magnesium supplementation on physical performance in healthy elderly women involved in a weekly exercise program: a randomized controlled trial. Am J Clin Nutr (2014) 100(3):974–81. 10.3945/ajcn.113.080168 25008857

[29] Matias CN Santos DA Monteiro CP Silva AM Raposo Mde F Martins F Magnesium and strength in elite judo athletes according to intracellular water changes. Magnes Res (2010) 23(3):138–41. 10.1684/mrh.2010.0217 20860960

[30] Moxham J Morris AJ Spiro SG Edwards RH Green M Contractile properties and fatigue of the diaphragm in man. Thorax (1981) 36(3):164–8. 10.1136/thx.36.3.164 7281080 PMC471468

[31] Anderson-Huntington RB Rosenblith JF. Central nervous system damage as a possible component of unexpected deaths in infancy. Dev Med Child Neurol (1976) 18(4):480–92. 955312 10.1111/j.1469-8749.1976.tb03687.x

[32] Flink EB. Magnesium deficiency. Etiology and clinical spectrum. Acta Med Scand Suppl (1981) 647:125–37. 7020347 10.1111/j.0954-6820.1981.tb02648.x

[33] Wong HB Teh YF An association between serum-magnesium and tremor and convulsions in infants and children. Lancet (1968) 2(7558):18–21. 10.1016/S0140-6736(68)92890-0 4172684

[34] Korobkin R Guilleminault C Neurologic abnormalities in near miss for sudden infant death syndrome infants. Pediatrics (1979) 64(3):369–74. 481982

[35] de Baaij JH Hoenderop JG Bindels RJ Magnesium in man: implications for health and disease. Physiol Rev (2015) 95(1):1–46. 10.1152/physrev.00012.2014 25540137

[36] Dallas RJ Cot death. Br Med J (1974) 2:348.4835839

[37] Ponsonby AL Dwyer T Gibbons LE Cochrane JA Jones ME McCall MJ. Thermal environment and sudden infant death syndrome: case-control study. BMJ (1992) 304(6822):277–82. 10.1136/bmj.304.6822.277 1739826 PMC1881052

[38] Kleemann WJ Schlaud M Poets CF Rothämel T Tröger HD. Hyperthermia in sudden infant death. Int J Legal Med (1996) 109(3):139–42. 10.1007/BF01369674 8956988

[39] Kahraman L Thach BT Inhibitory effects of hyperthermia on mechanisms involved in autoresuscitation from hypoxic apnea in mice: a model for thermal stress causing SIDS. J Appl Physiol (2004) 97(2):669–74. 10.1152/japplphysiol.00895.2003 15247199

[40] Stanton AN. Sudden infant death. Overheating and cot death. Lancet (1984) 2(8413):1199–201. 10.1016/S0140-6736(84)92753-3 6150244

[41] Fleming PJ Gilbert R Azaz Y Berry PJ Rudd PT Stewart A Interaction between bedding and sleeping position in the sudden infant death syndrome: a population based case-control study. BMJ (1990) 301(6743):85–9. 10.1136/bmj.301.6743.85 2390588 PMC1663432

[42] Editorial, prone, hot, and dead. Lancet (1990) 336(8723):1104. 1977987

[43] Doberentz E Führing S Madea B. Sudden infant death syndrome: no significant expression of heat-shock proteins (HSP27, HSP70). Forensic Sci Med Pathol (2016) 12(1):33–9. 10.1007/s12024-015-9730-4 26662848

[44] Haldane JS The influence of high air temperatures: no. 1. J Hyg (Lond) (1905) 5(4):494–513. 10.1017/S0022172400006811 20474239 PMC2236106

[45] Maskrey M. Influence of body temperature on responses to hypoxia and hypercapnia: implications for SIDS. Clin Exp Pharmacol Physiol (1995) 22(8):527–32. 10.1111/j.1440-1681.1995.tb02061.x 7586708

[46] Tsuji B Hayashi K Kondo N Nishiyasu T. Characteristics of hyperthermia-induced hyperventilation in humans. Temperature (Austin) (2016) 3(1):146–60. 10.1080/23328940.2016.1143760 27227102 PMC4879782

[47] Callahan LA Supinski GS. Sepsis induces diaphragm electron transport chain dysfunction and protein depletion. Am J Respir Crit Care Med (2005) 172(7):861–8. 10.1164/rccm.200410-1344OC 15994462

[48] Supinski GS Vanags J Callahan LA. Effect of proteasome inhibitors on endotoxin-induced diaphragm dysfunction. Am J Physiol Lung Cell Mol Physiol (2009) 296(6):L994–1001. 10.1152/ajplung.90404.2008 19376888 PMC2692801

